# Diagnostical Performances of the FilmArray Gastro-Intestinal Panel in Kidney Transplant Recipients

**DOI:** 10.3389/ti.2026.15766

**Published:** 2026-02-24

**Authors:** Elise Ferrie, Zoulkarnayni Nikiema, Arnaud Serret-Larmande, Noémie Simon-Tillaux, Nadia Arzouk-Snanoudj, Marta Cancella de Abreu, Brigitte Rached, Jérôme Robert, Pierre Galichon, Inna Mohamadou

**Affiliations:** 1 Kidney Transplant Service, Pitié-Salpêtrière Hospital, APHP.Sorbonne Université, Paris, France; 2 Public Health Department, PSL-CFX Clinical Research Unit, Pitié-Salpêtrière Hospital, APHP.Sorbonne Université, Paris, France; 3 Sorbonne University, INSERM, Pierre Louis Epidemiology and Public Health Institute, Paris, France; 4 Office of Biostatistics and Epidemiology, Gustave Roussy, Oncostat U1018, Inserm, University Paris-Saclay, labeled Ligue Contre le Cancer, Villejuif, France; 5 Emergency Department, GHU APHP-Sorbonne Université, Hôpital Pitié-Salpêtrière, APHP.Sorbonne Université, Paris, France; 6 Centre d’Immunologie et des Maladies Infectieuses - CIMI-Paris, Sorbonne Université, and Infection control Unit, Paris, France; 7 Bacteriology Department, Pitié-Salpêtrière Hospital, APHP.Sorbonne Université, Paris, France; 8 INSERM, Sorbonne Université, Common and Rare Kidney Diseases: from Molecular Events to Precision Medicine, CoRaKid, Paris, France

**Keywords:** diagnosis, diarrhea, kidney transplantation, PCR, performances

Dear Editors,

Diarrhea is a major burden for kidney transplant recipients (KTR), associated with dehydration, sepsis, acute kidney injury, immunosuppressor overdose or discontinuation, and rejection [1, 2]. Infections and drug toxicity are the main causes of diarrhea in KTR, with infection being present in 23%–51% of all cases [3–7]. Because of the variety of potential pathogens involved, it requires multiple and repeated stool samples for identification and appropriate treatment.

Diagnosis relies on microscopy, antigen detection, culture and specific polymerase chain reaction (PCR) [8]. They are time-consuming and can lack sensitivity. Multiplex PCR enables the diagnosis of a large range of microbiological agents in one unique sample, in a shorter timespan (around 1 h), and with a limited cost. FilmArray GastroIntestinal Panel (FAGIP) is a multiplex FDA-approved PCR, enabling the detection in stools of most pathogens involved in diarrhea. FAGIP has shown promising results in children [9], liver transplant recipients [10], and hematologic patients [11]. In KTR, data are limited and based on retrospective studies [12].

In this study, we compare the sensibility and specificity of FAGIP vs. standard tests in a population of KTR with diarrhea.

We performed a double-blind, observational, prospective cohort study in KTR from a single university hospital. The study was approved by the Institutional Review Board (IRB) CERC-MIT.

KTR hospitalized for acute diarrhea (<7 days) or who developed diarrhea during their hospital stay from April 2022 to February 2024 (with an interruption from May 2023 to October 2023 for the replacement of expired FAGIP kits and the relocation of the PCR device) were included in the study. The inclusion criteria consisted of adult KTR with a functional graft who did not express opposition to the study. Exclusion criteria were patients with a non-functional kidney transplant, resolution of diarrhea before samples could be achieved, those already included in the study for a diarrhea episode, and patients with a known positive CMV-PCR in blood tests during the 7 days preceding the study, as the diagnosis of CMV-related diarrhea does not rely on stool tests. Patients were included prospectively at their hospital admission and followed throughout their entire hospital stay.

Standard tests were prescribed by the attending physician following the procedures of each local laboratory: (i) bacteriological cultures for classical digestive pathogens (*Salmonella* spp., *Shigella* spp., *Campylobacter* spp., *Yersinia* spp.) and enzyme immunoassay for shiga-toxin in case of bloody stools; (ii) tests for toxinogenic *C. difficile*; (iii) tests for enteropathogenic parasites (cryptosporidium or microsporidium) in the parasitology department (three stool samples within 5 days); and (iv) tests for enteric viruses (adenovirus, enterovirus, sapovirus, or norovirus) in the virology department.

FAGIP was performed on a stool sample by an independent clinical study technician, blinded from the patient, the circumstances of diarrhea, and the results of standard analyses. Investigators and clinicians were blinded from the FAGIP results in order not to interfere with the management of the patients.

Clinical and biological data were prospectively collected from electronic medical files.

Thirty-four patients were included in the study. Clinical and microbiological characteristics are summarized in Table 1. The median age was 51 years. Immunosuppression included corticosteroid (100%), mycophenolate mofetil (97.1%), or tacrolimus (97.1%). The median time between transplantation and diarrhea was 6.1 months. Twenty-three (67.6%) patients had Acute Kidney Injury and 55.9% had intravenous hydration. The median serum creatinine was 2.79 mg/dL.

**TABLE 1:** Population’s characteristics and FAGIP results.

Population’s characteristics		Median [IQT] or number (percentage)
Age (median, IQT)		51.5 [43.3; 60.7]
Renal graft alone (n,%)		34 (100%)
Immunosuppressive treatment (n, %)		
Corticoids		34 (100%)
Mycophenolate mofetil		33 (97.1%)
Tacrolimus		33 (97.1%)
Ciclosporine		1 (2.9%)
mTORi		1 (2.9%)
Delay since graft (months) (median, IQT)		6.1 [1.17; 8.4]
Hospitalization (n,%)		26 (76%)
Length of hospitalization (days, median, IQT)		7 [2.0; 16.7]
Fever (n, %)		23 [67.6%]
IV hydration (n,%)		15 (44.1%)
Length of hydration (days, median, IQR)		4.5 [3.25; 6.75]
Acute kidney injury (n, %)		23 (34.6%)
Initial creatininemia (mg/L) (median, IQR)		2.79 [1.8; 4.08]
CRP (mg/L) (median, IQR)		4.02 [1.09; 20.74]
Stool samples (n, %)		
Standard stool culture		30 (88%)
Search for *C. difficile* toxin		34 (100%)
Parasitologic		31 (91%)
Virologic		9 (26%)
Delay before diagnostic (days, median, IQT)		6 [3; 9,7]
Delay before specific treatment (days, median, IQT)		7 [6; 9,5]
FAGIP and gold standard results		
Positive FAGIP		13 (38.2%)
- Positive FAGIP and positive gold standard - Positive FAGIP and negative gold standard		4 9
Negative FAGIP		21 (61.8%)
- Negative FAGIP and negative gold standard - Negative FAGIP and positive gold standard		19 2
Comparison between pathogens identified with FAGIP and gold standard		
FilmArray GI panel		
Pathogen 1	Pathogen 2	Gold standard
Enteropathogenic E.coli	*Campylobacter*	​
Enteropathogenic E.coli	*C. difficile*	-
Shiga-like toxin-producing E.coli	Cryptosporidium	Enteropathogenic E.coli
Cryptosporidium	-	Cryptosporidium
Sapovirus	-	Cryptosporidium
Enteroaggregative E.coli	-	-
Enteropathogenic E.coli	-	-
Enteropathogenic E.coli	-	-
Enteropathogenic E.coli	Enterotoxigenic E.coli	-
Enteropathogenic E.coli	Cryptosporidium	-
Enteroinvasive E.coli	*Shigella*	Cryptosporidium
Rotavirus	-	-
Sapovirus	-	-

Amongst 34 patients, 30 (88.2%) had a stool culture and 31 (91.1%) a parasitological stool test, but only 9 (26.5%) had a virological test. FAGIP was performed in all patients. Antimicrobial treatment for diarrhea was initiated in 4/34 (11.8%) patients, with a delay of 5 days.

Six (17.6%) patients had a diagnosis of infectious diarrhea (Table 1). In two of them, the diagnosis was made in spite of negative stool tests: one patient had a blood culture positive for *Klebsiella pneumoniae* and *E.coli* with a negative stool culture, and the second patient was diagnosed with CMV disease. FAGIP was negative in these two patients.

In the four patients with a positive standard test, FAGIP was also positive and identified the same pathogen. In three cases, FAGIP detected a second pathogen (Table 1). In the 30 patients with negative standard tests, FAGIP was positive in nine patients. Altogether, FAGIP was positive in 13 out of 34 patients (38.2%).

To investigate the significance of a positive FAGIP with negative standard tests, we studied clinical and demographic features: systolic arterial blood pressure (BP) was lower in patients with a positive FAGIP (median 121 vs. 138 mmHg, p = 0.049). Other features including temperature, kidney function, and length of stay were not significantly different.

These results highlight several key findings:

1. In our cohort of 34 patients, standard methods had limited yield since they could identify pathogens in only 4/34 (11.7%) patients (three parasites and one bacteria). The virological test could be performed in only 26.5% of patients, underscoring the difficulty of performing repeated stool sampling in real-life settings.
2. FAGIP was concordant with standard methods in the four patients with a positive standard test.
3. FAGIP was positive in 9/34 patients with negative standard tests (26.5%).

In these nine patients, FAGIP positivity with negative standard tests could have warranted a specific treatment or modification of immunosuppressive regimen. However, it is not possible at this stage to distinguish asymptomatic carriage from authentic active infection (as suggested by the slightly lower BP). Eventually, all these patients evolved well without a specific treatment. Therefore, it is also possible that FAGIP results were falsely positive, by detecting DNA or RNA of pathogens that can persist in stool after healing an infection or colonizing non-pathogenic agents.

An interventional study is thus needed to assess the effect of treating patients based on FAGIP results. Resolution of diarrhea, but also health costs and antibiotic resistance, are relevant outcomes, since the integration of highly sensitive multiplex PCR tests generally leads toward more antibiotics and anti-viral and anti-parasitic drug use.

In conclusion, FAGIP is an easy-to-implement and sensitive method for the identification of diarrhea-associated pathogens in KTR. Its increasing use in place of the standard microbiology tests raises concerns about overdiagnosis and overtreatment that will need further prospective evaluation.

## Data Availability

The original contributions presented in the study are included in the article/supplementary material, further inquiries can be directed to the corresponding authors.
